# Improvement of Adaptive GAs and Back Propagation ANNs Performance in Condition Diagnosis of Multiple Bearing System Using Grey Relational Analysis

**DOI:** 10.1155/2014/419743

**Published:** 2014-12-18

**Authors:** Lili A. Wulandhari, Antoni Wibowo, Mohammad I. Desa

**Affiliations:** ^1^School of Computer Science, Bina Nusantara University, Jakarta 11480, Indonesia; ^2^School of Quantitative Sciences, UUM College of Arts and Sciences, Universiti Utara Malaysia, 06010 Sintok, Kedah, Malaysia; ^3^Advanced Informatics School (AIS), Universiti Teknologi Malaysia, 54100 Kuala Lumpur, Malaysia

## Abstract

Condition diagnosis of multiple bearings system is one of the requirements in industry field, because bearings are used in many equipment and their failure can result in total breakdown. Conditions of bearings commonly are reflected by vibration signals data. In multiple bearing condition diagnosis, it will involve many types of vibration signals data; thus, consequently, it will involve many features extraction to obtain precise condition diagnosis. However, large number of features extraction will increase the complexity of the diagnosis system. Therefore, in this paper, we presented a diagnosis method which is hybridization of adaptive genetic algorithms (AGAs), back propagation neural networks (BPNNs), and grey relational analysis (GRA) to diagnose the condition of multiple bearings system. AGAs are used in the diagnosis algorithm to determine the best initial weights of BPNNs in order to improve the diagnosis accuracy. In addition, GRA is applied to determine and select the dominant features from the vibration signal data which will provide good diagnosis of multiple bearings system in less features extraction. The experiments results show that AGAs-BPNNs with GRA approaches can increase the accuracy of diagnosis in shorter processing time, compared with the AGAs-BPNNs without the GRA.

## 1. Introduction

A bearing is a device widely used in industries to minimize friction on rotating part of machine by giving smooth metal balls or roller and a smooth inner and outer metal surface for the balls to roll against. Unfortunately, bearing is one of the machine parts which has high percentage of failures compared to other components [1]. Based on previous study, bearings contribute 40–50% causes of machine failure [2]. Therefore, a precise condition diagnosis of bearing system is essential to detect defects before they lead to failures.

**Figure 1:**
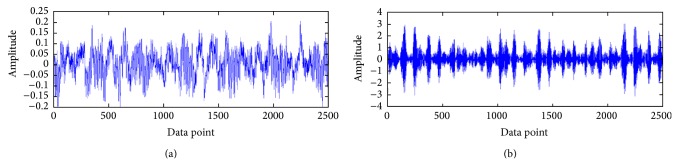
Vibration signal data of normal bearing (a) and faulty bearing (b).

A precise condition diagnosis can be achieved by good condition monitoring. Condition monitoring of a bearing is reflected by vibration signal data. The vibration signal data is captured by accelerometers which record the condition of the bearing continuously. Vibration signal data is commonly used for bearing condition diagnosis due to its intrinsic advantage of revealing bearing failure [3–5]. Vibration signal under different condition will show different pattern [6], as can be seen in Figure 1. Figure 1 shows that the vibration signal data of normal bearing has different pattern from faulty bearing. Vibration signal of faulty bearing has higher amplitude compared with the normal bearing. However, multiple bearing system with one faulty bearing may not be visually different on the represented vibration signal data compared to all bearings being normal. Therefore, it is important to have a technique which is able to accurately diagnose the system condition based on the continuously monitored vibration signals.

It is important to extract features from vibration signal data, since the vibration signal data captured from mechanical system such as bearing are complex in nature and some of the useful information is corrupted [5, 7]. In order to capture the diagnostic information from the vibration signal data, it is appropriate to compute as many features as possible. In this paper, we extract ten features from vibration signal data of the bearing system. Those features are standard deviation, skewness, kurtosis, the maximum peak value, absolute mean value, root mean square value, crest factor, shape factor, impulse factor, and clearance factor [8]. These features are effective and practical for condition diagnosis due to their relative sensitivity to early faults and robustness to various loads and speeds [9]. But the choice of features is often arbitrary, which will lead the situation where some features contain the same information and the others contain no useful information at all [8]. Therefore, a technique to determine which are the dominant features for multiple bearing diagnoses is important. One of the simplest ways to find the dominant parameters is by trying and combining the features continuously up to the desired result achieved. However, this method will spend much time and memory to obtain the dominant features. Grey relational analysis (GRA) is one of the methods which are commonly used to find the dominant features. It is used as feature selection method to remove irrelevance and redundant factors that affect the results [10]. The essential thing of GRA is that it can be used to describe the relation among features [11]. GRA can be employed to explain the complicated interrelationship among the data when the trends of their development are either homogeneous or heterogeneous [12]. In this study, we used grey relational analysis (GRA) to determine the dominant features which contain useful information in multiple bearings condition diagnosis. The dominant features from GRA will be used as the input in condition diagnosis algorithm.

Diagnosis algorithm had been proposed by many researchers, some of them using individual metaheuristic techniques such as the genetic algorithms (GAs) and fuzzy and neural networks (NNs) [13–17]. However, individual metaheuristic techniques suffer from their own drawbacks, which can be overcome by forming a hybrid approach combining the advantages of each technique [18]. Hence, researchers have recently started to propose hybrid metaheuristic techniques to improve the performance of condition diagnosis. Wulandhari et al. [19–21] improved the condition diagnosis work in specific type of fault for multiple bearing using hybrid genetic algorithms and back propagation neural networks (GAs-BPNNs) approach. In this paper, we propose an improvement of GA-BPNNs by applying adaptive methods to GAs and back propagation neural networks (AGAs-BPNNs) and using GRA to identify and select the dominant features for AGAs-BPNNs algorithm in order to obtain a precise condition diagnosis for the multiple bearing systems.

## 2. Bearing Vibration Signal Data

**Figure 2:**
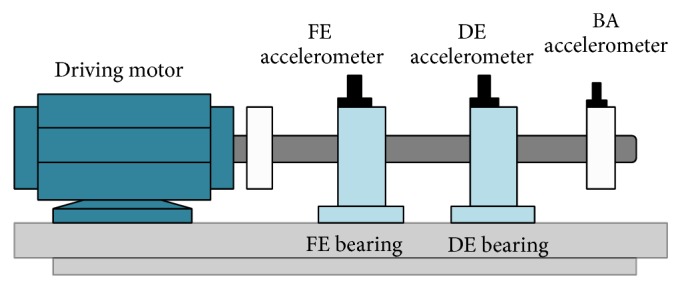
Bearing and accelerometer structure.

In this paper, we use the vibration signals data from the Case Western Reserve University Bearing Data Center [22]. The vibration signals data were captured from a two-bearing system, which consists of Drive End bearing (DE) and Fan End bearing (FE), with various combinations of the bearing conditions. The specifications of the bearing are given in Table 1. For the purpose of capturing the vibration signals data, three accelerometers were attached on the bearings and the baseline (BA) as shown in Figure 2. Bearing vibration data were collected under seven different conditions: (1) FE and DE Normal, (2) FE Normal and DE Inner Race Fault (DE-IRF), (3) FE Normal and DE Ball Fault (DE-BF), (4) FE Normal and DE Outer Race Fault (DE-ORF), (5) FE Inner Race Fault (FE-IRF) and DE Normal, (6) FE Ball Fault (FE-BF) and DE Normal, and (7) FE Outer Race Fault (FE-ORF) and DE Normal. The example of the data captured is presented in Table 2.

From Table 2, we can see that each condition has three streams of data as captured by the three accelerometers; thus, each feature will be extracted from three accelerometers. Based on the available data, generally only seven condition classes of bearing can be specified as the output of the diagnosis. In this paper, we expanded the condition classes from seven to sixteen classes by combining and mixing the available data. For the FE-IRF and DE-IRF class, for instance, its BA data was set or obtained from the average of BA accelerometer in FE-IRF and DE-IRF condition, the FE data was obtained from FE accelerometer in FE-IRF condition, and the DE data was obtained from DE accelerometer in DE-IRF condition. The expansion of condition classes was done to obtain more specific condition diagnosis for each bearing so that any action to each bearing can be specifically carried out. The advantage of this expansion is that, here, we can identify the condition of DE and FE bearing simultaneously. In the seven classes case, we can only identify the condition of either one. The sixteen classes of the bearing conditions are presented in Table 3.

The classes of multiple bearing conditions are influenced by ten features extracted from the data. The values of the features lie within the interval which is the lower and upper bound of the data extraction. The interval of the features values is presented in Table 4.

## 3. The Proposed Algorithm

**Figure 3:**
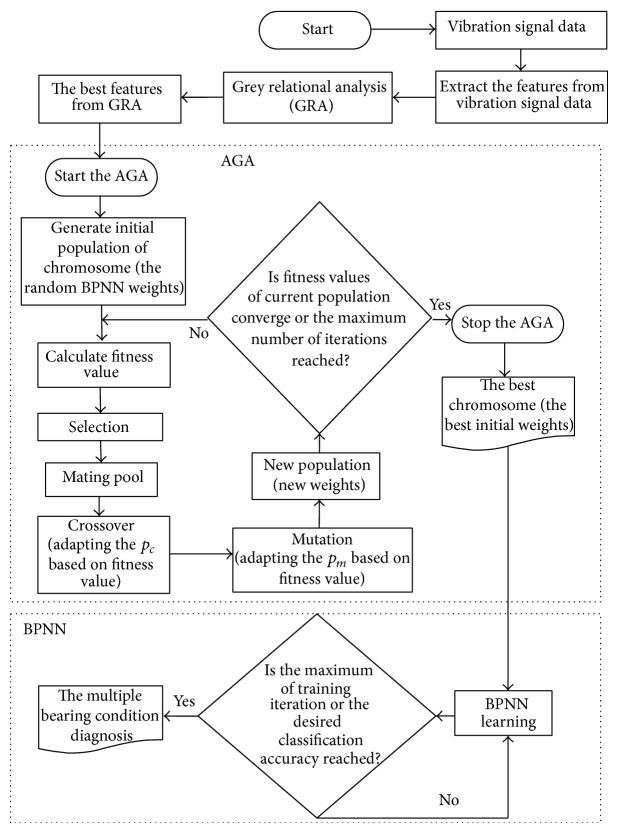
The framework of GRA-AGAs-BPNNs.

This paper proposed a hybrid method of GRA, AGA, and BPNN to diagnose the condition of multiple bearing systems. This hybridization applies GRA to determine the dominant features by analyzing the relation between each feature and its ideal values. The algorithm is started by initializing the features which are extracted from the vibration signal data and then calculate the grey relational coefficient (GRC) followed by calculating the grey relational grade (GRG) which both of them including to the GRA process. The results from the GRA are the dominant features which will be used as the input of the AGAs-BPNNs. The framework of the GRA-AGA-BPNNs is shown in Figure 3.

### 3.1. Grey Relational Analysis (GRA)

**Figure 4:**
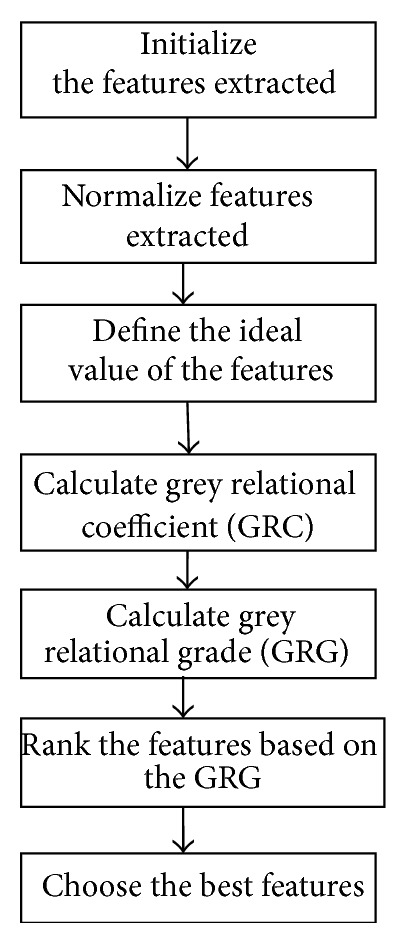
Grey relational analysis procedures.

A system for which the relevant information is completely known is called a white system, while a system for which the relevant information is completely unknown is a black system. Any system between these limits is a grey system which has poor and limited information [23]. In multiple bearing systems, any information about the condition of the bearing is not completely revealed by the features extracted from the vibration signal data. This unclear condition of data can be overcome using GRA which was proposed by Deng [24] in 1982. GRA utilizes the mathematical method to analyzing correlation between the references series which is the ideal value of features and the alternatives series [25]. It firstly normalizes the features extracted and then translates the performance of all alternatives into a comparability sequence with the ideal value called grey relational generating [26], followed by the calculation of grey relational coefficient between all comparability sequences and the references sequences. Finally, the grey relational grade between the reference sequence and every comparability sequence is calculated based on the grey relational coefficient. The highest grey relational grade of the alternatives features indicates that the features have dominant influence to the condition diagnosis. The procedures of GRA are shown in Figure 4.

The GRA starts by normalizing the features extracted, for the simplification; we omit the units of all parameters. This can be done easily by multiplying the certain parameter, say *p*_*i*_, with 1/the unit of parameter *p*_*i*_. Let *x*_*ij*_ be the *i*th sample of the *j*th feature extracted where *i* = 1,2,…, 240 and *j* = 1,2,…, 10:

(1) $$\begin{array}{c} x_{\max}=\max\left(x_{ij}\;|\;i=1,2,\ldots ,240;j=1,2,\ldots ,10\right),\\ x_{\min}=\min\left(x_{ij}\;|\;i=1,2,\ldots ,240;j=1,2,\ldots ,10\right). \end{array}$$

Then, the normalized features *x*_ij_^*^ can be obtained by the following equation [27]:

(2) $$\begin{array}{c} x_{ij}^{*}=\frac{x_{ij}-x_{\min}}{x_{\max}-x_{\min}}. \end{array}$$

Next, the grey relational generating is conducted by determining the reference or ideal values of the features extracted. Let *P*_*j*_ be the features extracted, with *j* = 1,2,…, 10. *C*_*s*_ is the condition of the bearing systems with *s* = 1,2,…, 16. It is noticed that we used 240 pieces of sample data and 16 classes for bearing conditions where each class *C*_*s*_ consists of 15-item data which fall in the condition class *s*. The ideal values of the features extracted, say *z*_*sj*_, are the average of *j*th feature extracted value in *s*th condition which can be written as follows:

(3) $$\begin{array}{c} z_{sj}=\frac{1}{15}\overset{15s}{\underset{i=15s-14}{\sum }}x_{ij}^{*}. \end{array}$$

For example, *z*_11_ is the ideal value for the first condition class (class of FE and DE Normal) and the parameter *p*_1_. Regarding obtaining the next step, namely, grey relational coefficient, we need to determine the comparability sequences of the alternatives values and the ideal values. It is noticed that we used 240 pieces of sample data and 16 classes for bearing conditions where each class *C*_*s*_ consists of 15-item data which fall in the class *s*. Then, we define

(4) wij=z1j for  j=1,…,10;  i=1,…,15,wij=z2j for  j=1,…,10;  i=16,…,30,wij=z3j for  j=1,…,10;  i=31,…,45,    ⋮wij=znj for  j=1,…,10;  i=15n−1+1,…,15n,

where *n* = 1,…, 16; then, the comparability of the alternatives and ideal values can be calculated as

(5) $$\begin{array}{c} \Delta w_{ij}=\left|x_{ij}^{*}-w_{ij}\right|, \end{array}$$

where Δ*w*_*ij*_ is the comparability of alternatives and ideal values, *x*_ij_^*^ is the alternatives value which is normalized features extracted from the vibration signal data, and *w*_*ij*_ is the ideal values which is defined based on the condition classes. Based on (4) and (5), the next step of GRA procedures, namely, grey relational coefficient calculation between comparability sequences and ideal sequences, is written as follows:

(6) GRCij=min⁡k⁡min⁡l⁡Δwkl+ξmax⁡k⁡max⁡l⁡ΔwklΔwij+ξmax⁡k⁡max⁡l⁡Δwijfor  k=1,2,…,240;  l=1,2,…,10,

where GRC_*ij*_ is the grey relational coefficient value of *i*th samples and *j*th feature and *ξ* is the distinguishing coefficient which is defined in the range 0 ≤ *ξ* ≤ 1. Then, the grey relational grade (GRG) is determined by averaging the GRC to each feature and is represented as

(7) $$\begin{array}{c} \text{GR}{\text{G}}_{j}=\frac{1}{m}\overset{m}{\underset{i=1}{\sum }}\text{GR}{\text{C}}_{ij}, \end{array}$$

where *m* is the number of samples. Equation (7) is used to find the GRG of accelerometers BA, DE, and FE. The final grey relational grade on the feature *j*, say GRG_*j*_, is the average of GRG_j_^*^s from BA, DE, and FE, respectively. The results of the experiment using GRA are presented in Section 4.

### 3.2. The Proposed GRA-AGAs-BPNNs Algorithm

The proposed GRA-AGAs-BPNNs algorithm is the hybrid algorithm which combines the advantages of GRA, GAs, and BPNNs to one algorithm for the condition diagnosis of the multiple bearing systems. The dominant features from GRA are used in AGAs-BPNNs algorithm to classify the condition effectively. Adaptive operator probabilities techniques in GAs are proposed to obtain better initial weights for BPNNs training. The adaptive technique is applied to maintain the diversity of the population by varying the probabilities of crossover (*p*_*c*_) and mutation (*p*_*m*_) as, for example, [28–31]. The algorithm for the proposed AGAs-BPNNs is as follows.

(1) Let (*I*_*k*_, *T*_*k*_) be the *k*th input and target pair of the problem to be solved by BPNN, with *k* = 1,2,…, *N*_in_, and *N*_in_ is the number of paired data.
(2) Let *N*_pop_, *N*_chro_, *p*_*c*0_, *p*_*m*0_, and *N*_iter_ be the number of populations, number of chromosomes, initial crossover probability, initial mutation probability, and maximum number of iterations, respectively. Initialize *p*_*c*0_, *p*_*m*0_, *R*_*pc*_, and *R*_*pm*_ where *R*_*pc*_ are random vectors of numbers which generated in range [0,1] with size 1 × *N*_chro_/2 and *R*_*pm*_ are random vectors of numbers which generated in range [0,1] with size 1 × *N*_chro_. Set *i* = 0.
(3) Determine the number of dominant features chosen.
(4) Determine the BPNN architecture in terms of the number of input neuron, hidden layer, hidden neuron and output neuron, and the activation functions based on the number of dominant features chosen.
(5) Generate an initial population of chromosomes *Q*_0_. Each chromosome contains genes which correspond to BPNN random weights, and the number of genes in a chromosome is equal to the number of BPNN weights.
(6) Calculate the fitness value *F*(*i*, *j*) of the *j*th chromosome in population *Q*_*i*_ using
  (8) $$\begin{array}{c} F\left(i,j\right)=\frac{1}{E\left(i,j\right)},\;j=1,2,\ldots ,N_{\text{chro}}, \end{array}$$
 where *E*(*i*, *j*) is the mean square error (MSE) of the *j*th chromosome in the population *Q*_*i*_. It is calculated based on the selected BPPN architecture as follows:
  (9) $$\begin{array}{c} E\left(i,j\right)=\frac{1}{2}\overset{N_{\text{in}}}{\underset{k=1}{\sum }}{\left(T_{kj}-O_{kj}^{i}\right)}^{2}, \end{array}$$
 where *T*_*kj*_ is the target of the *k*th input in the *j*th chromosome and *O*_*kj*_^*i*^ is the output of the *k*th input in the *j*th chromosome of the population *Q*_*i*_ based on the selected BPNN architecture.
(7) Generate the mating pool by selecting the best chromosomes using roulette selection methods.
(8) Select parent pairs of population *Q*_*i*_, say (*ϕ*_1*s*_^*i*^, *ϕ*_2*s*_^*i*^), from the mating pool for crossover mechanism where *s* = 1,2,…, *S* and *S* = ⌊*N*_chro_/2⌋.
(9) Calculate the crossover probability of the *s*th parents pairs in the population *Q*_*i*_ [32]:
  (10) pc(i,ϕ1si,ϕ2si) =pc0Fmax⁡i−F′i,sFmax⁡i−F¯iif  F′i,s>F¯ipc0otherwise,whereF′(i,s)=Fϕ1siif  F(ϕ1si)>F(ϕ2si)Fϕ2siotherwise.
 
*F*(*ϕ*_1*s*_^*i*^) and *F*(*ϕ*_2*s*_^*i*^) are the fitness values of parent 1 and parent 2, respectively; *F*_max⁡_(*i*) is the maximum fitness value of the population *Q*_*i*_; $\bar{F}(i)$ is the average fitness value of the population *Q*_*i*_.
(10) Calculate mutation probability of the *j*th chromosome in the population *Q*_*i*_ [32]:
  (11) pm(i,j)=pm0Fmax⁡(i)−F(i,j)Fmax⁡(i)−F¯(i)if  F(i,j)>F¯(i)pm0otherwise,

 where *F*(*i*, *j*) is the fitness value of the *j*th chromosome in the population *Q*_*i*_.
(11) Set *i* = *i* + 1.
 Generate *Q*_*i*_ by applying crossover and mutation mechanism based on the following rules.
  - For *s* = 1 : *S*,
  - if *p*_*c*_(*i*, *ϕ*_1*s*_^*i*^, *ϕ*_2*s*_^*i*^) > *R*_*pc*_(*s*), do a crossover between *ϕ*_1*s*_^*i*^ and *ϕ*_2*s*_^*i*^. Otherwise, copy *ϕ*_1*s*_^*i*^ and *ϕ*_2*s*_^*i*^ as offspring.
  - For *j* = 1 : *N*_chro_,
  - if *p*_*m*_(*i*, *j*) > *R*_*pm*_(*j*), do a mutation of the *j*th chromosome. Otherwise, the *j*th chromosome is kept unchanged.
(12) If *Q*_*i*_ converge or *i* is equal to *N*_iter_, then the best chromosome is obtained and used as the initial weights for BPNN learning. Otherwise, go to step (6).

## 4. Experimental Evaluation and Discussion 

In this section, we will describe and discuss the result of the experiment from GRA-AGAs-BPNNs in classifying the condition of the multiple systems. For the GRA process, we tried several values of *ξ*, namely, 0.3, 0.5, 0.6, and 1. Based on (7), GRG value for each was calculated. The sequence of the features extracted based on GRG value is given in Table 5.

**Figure 5:**
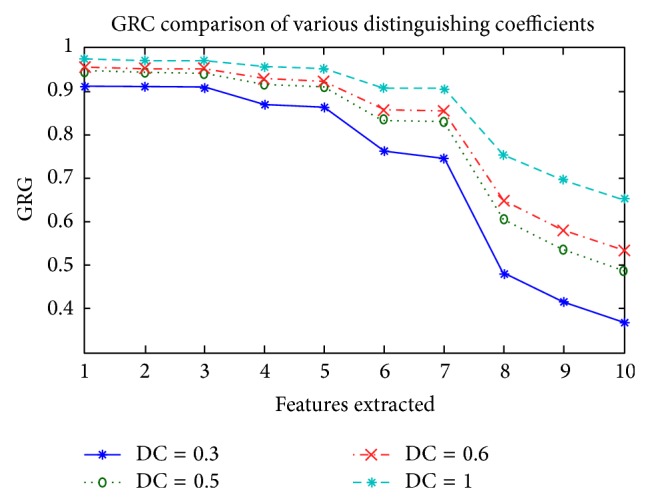
GRG comparison of various distinguishing coefficients.

Table 5 shows that if the distinguishing coefficient *ξ* is closer to 0, then the GRG of the feature will have a range wider than if the distinguishing coefficient *ξ* is closer to 1. For *ξ* = 0.3, the GRG range is around 0.545 while the *ξ* = 1 GRG is around 0.320 as shown in Figure 5. From Table 5, we can see, however, that the *ξ* values are varied and the sequence of features extracted is the same. Root mean square value is the features with the highest GRG value.

In this paper, we conducted experiments of AGAs-BPNNs using one, three, five, and seven of the best dominant features based on the GRG values in Table 5. In AGAs-BPNNs techniques, we set AGAs features as follows: 100 chromosomes of each population, initial crossover probability of 0.6, and initial mutation probability of 0.01. For BPNNs, we use three hidden layers and refer to *m*-*l*_1_-*l*_2_-*l*_3_-*n* as *m* neurons input, *l*_1_ neurons in the first hidden layers, *l*_2_ neurons in the second hidden layers, *l*_3_ neurons in the third hidden layers, and *n* neurons output. As stated in Section 2, vibration signals data is recorded from three accelerometers; thus, features are extracted from three accelerometers which cause the number of neuron inputs in BPNNs to be equal to 3 × number of features. For example, if we choose five dominant features, then the neuron input contains 15 neuron inputs, where each neuron contains 240 samples of data, while each hidden layer contains 15 neurons as well and 16 neurons in output layer. The 240 samples of data are split randomly into three sets: 80% for training, 10% for validation, and 10% for testing. BPNN uses* logsig* and* purelin* as the activation function, where learning rate and momentum are 0.05 and 0.25, respectively.

We conducted ten times the experiments of the GRA-AGAs-BPNNs for each one, three, five, and seven dominant features combination. We also conducted experiments using the lowest GRG features and combination of the highest and the lowest GRG features in AGA-BPNN to obtain condition diagnosis of multiple bearing systems. These experiments were carried out to see the influence of the selection of dominant features combination on condition diagnosis algorithm performance for multiple bearing systems. The performance of the algorithm is characterized based on the accuracy in classifying the condition of the bearings. The classification accuracy is computed by

(12) classification  accuracy =total  true  predicted  classtotal  output×100%.

The experiments were executed using a computer with Intel Core 2 Quad processor Q8200, 2.33 GHz and 1.96 GHz, and RAM 3.46 GB. The result of the GRA-AGAs-BPNNs is given in Table 6.

Table 6 shows the comparison of the classification accuracy between GRA-AGAs-BPNNs and AGA-BPNN algorithm without GRA (which involve ten features). We can see that the classification accuracy of GRA-AGA-BPNN using five and seven dominant features with topologies 15-15-15-15-16 and 21-21-21-21-16, respectively, has higher accuracy than AGA-BPNN with topology 30-30-30-30-16 without GRA and using ten features. From the experiment results, we obtain that root mean square value, standard deviation, absolute mean value, skewness, and maximum peak value can give good diagnosis of multiple bearing conditions. We notice that, by applying GRA in AGA-BPNN algorithm, we can achieve higher classification accuracy with shorter time. GRA is capable of determining which features can give dominant contribution in condition diagnosis of the multiple bearing system.

## 5. Conclusion

In this paper, we introduced a new hybrid technique of grey relational analysis, adaptive operator probabilities in genetic algorithms (AGAs), and back propagation neural networks (BPNNs), called GRA-AGAs-BPNNs for condition diagnosis of multiple bearing systems. We used grey relational analysis (GRA) to determine the dominant features which contain useful information of multiple bearings condition. GRA determines that mean square value, standard deviation, absolute mean value, skewness, and maximum peak value can give good diagnosis of the multiple bearings condition. The features from GRA are used in AGA-BPNNs to diagnose the condition effectively. We exploited the strong capability in optimization of genetic algorithms, which here have been further improved by varying the mutation and crossover operators probabilities, for searching the best initial weights for BPNNs, and the strong capability in classification of BPNNs to classify or diagnose the condition of a multiple bearing system. The AGAs strengthen the BPNNs to achieve the higher classification accuracy in shorter CPU time compared to the standard BPNN or the hybrid GAs-BPNNs.

Experimental results showed that the GRA is capable of improving the classification accuracy of AGAs-BPNNs in shorter time. The accuracy achieves 100%, 94.6%, and 92.5% for the training, validation, and testing, respectively, for 7 dominant features and 99.4%, 97.1%, and 96.7% for the training, validation, and testing, respectively, for 5 dominant features. The accuracy is increased and the processing time is reduced compared to the AGAs-BPNNs without GRA as shown in Table 7. This achievement provides the benefits for condition diagnosis in the real case, since we require a precise and quick process to diagnose the condition of multiple bearing in order to avoid total breakdown.

## Figures and Tables

**Table 1:** Multiple bearings specifications [22].

Bearing	Inside diameter (inches)	Outside diameter (inches)	Thickness (inches)	Ball diameter (inches)	Pitch diameter (inches)
DE bearing	0.9843	2.0472	0.5906	0.3126	1.537
FE bearing	0.6693	1.5748	0.4724	0.2656	1.122

**Table 2:** Example of bearing vibration data.

	Normal		Drive end bearing fault data									Fan end bearing fault data								
Number	bearing		Inner race fault			Ball fault			Outer race fault			Inner race fault			Ball fault			Outer race fault		
	DE	FE	BA	DE	FE	BA	DE	FE	BA	DE	FE	BA	DE	FE	BA	DE	FE	BA	DE	FE
1	0.053	0.146	0.065	−0.083	−0.402	0.016	−0.003	−0.247	0.000	0.009	−0.407	0.098	−0.025	−0.051	0.017	−0.168	0.320	−0.031	−0.134	0.127
2	0.089	0.098	−0.023	−0.196	−0.005	0.017	−0.096	0.143	0.069	0.424	0.263	0.042	−0.029	−0.192	−0.004	0.181	0.326	−0.120	0.003	−0.259
3	0.100	0.055	−0.089	0.233	−0.107	−0.036	0.114	0.003	0.031	0.013	0.495	−0.042	−0.046	0.051	−0.169	0.044	−0.260	−0.006	−0.027	−0.060
4	0.059	0.037	−0.094	0.104	−0.074	−0.045	0.257	−0.107	−0.037	−0.265	−0.423	0.081	0.001	0.151	−0.069	−0.270	0.031	0.060	−0.184	0.454
5	−0.005	0.054	−0.076	−0.181	0.209	0.008	−0.058	0.136	−0.116	0.237	−0.307	0.059	−0.037	−0.095	0.090	−0.138	0.447	−0.131	−0.203	0.075

**Table 3:** Sixteen classes of bearing conditions.

Number	Condition
C1	FE and DE Normal
C2	FE Normal and DE-IRF
C3	FE Normal and DE-ORF
C4	FE Normal and DE-BF
C5	FE-IRF and DE Normal
C6	FE-ORF and DE Normal
C7	FE-BF and DE Normal
C8	FE-IRF and DE-IRF
C9	FE-IRF and DE-ORF
C10	FE-IRF and DE-BF
C11	FE-ORF and DE-IRF
C12	FE-ORF and DE-ORF
C13	FE-ORF and DE-BF
C14	FE-BF and DE-IRF
C15	FE-BF and DE-ORF
C16	FE-BE and DE-BF

**Table 4:** The proposed feature interval of the sixteen condition classes.

Number	Condition classes	*p*_1_	*p*_2_	*p*_3_	*p*_4_	*p*_5_	*p*_6_	*p*_7_	*p*_8_	*p*_9_	*p*_10_
C1	DE and FE Normal	[0.064, 0.088]	[−0.166, 0.336]	[2.361, 3.421]	[0.185, 0.291]	[0.054, 0.073]	[0.005, 0.008]	[29.942, 47.827]	[0.083, 0.114]	[2.966, 4.524]	[3.471, 5.279]
C2	FE Normal and DE IRF	[0.087, 0.094]	[−0.135, 0.247]	[2.831, 3.497]	[0.236, 0.330]	[0.068, 0.075]	[0.008, 0.009]	[30.717, 38.750]	[0.110, 0.119]	[3.465, 4.586]	[4.182, 5.487]
C3	FE Normal and DE ORF	[0.078, 0.093]	[−0.256, 0.009]	[2.636, 3.390]	[0.223, 0.368]	[0.062, 0.074]	[0.006, 0.009]	[29.835, 45.121]	[0.097, 0.117]	[3.482, 5.063]	[4.123, 6.018]
C4	FE Normal and DE BF	[0.033, 0.039]	[−0.131, 0.171]	[2.688, 3.380]	[0.099, 0.139]	[0.026, 0.031]	[0.001, 0.002]	[76.682, 104.733]	[0.192, 0.230]	[3.486, 4.797]	[4.087, 5.650]
C5	FE IRF and DE Normal	[0.063, 0.092]	[−0.191, 0.153]	[2.202, 5.258]	[0.198, 0.276]	[0.036, 0.077]	[0.004, 0.009]	[24.479, 55.206]	[0.103, 0.113]	[2.609, 5.978]	[2.975, 13.998]
C6	FE ORF and DE Normal	[0.083, 0.085]	[−0.114, 0.035]	[2.383, 2.672]	[0.199, 0.238]	[0.068, 0.070]	[0.006, 0.007]	[27.955, 33.393]	[0.101, 0.104]	[2.878, 3.458]	[3.358, 4.032]
C7	FE BF and DE Normal	[0.117, 0.121]	[−0.139, 0.038]	[1.978, 2.342]	[0.239, 0.319]	[0.099, 0.104]	[0.014, 0.015]	[17.123, 22.767]	[0.137, 0.144]	[2.343, 3.213]	[2.613, 3.671]
C8	FE IRF and DE IRF	[0.077, 0.092]	[−0.149, −0.014]	[2.579, 4.377]	[0.228, 0.286]	[0.054, 0.075]	[0.006, 0.009]	[28.882, 46.978]	[0.107, 0.114]	[3.173, 5.282]	[3.726, 9.742]
C9	FE IRF and DE ORF	[0.078, 0.093]	[−0.175, −0.014]	[2.441, 4.231]	[0.232, 0.309]	[0.051, 0.075]	[0.005, 0.009]	[29.705, 47.740]	[0.101, 0.114]	[3.280, 5.038]	[3.833, 9.406]
C10	FE IRF and DE BF	[0.046, 0.064]	[−0.151, 0.089]	[2.460, 4.083]	[0.157, 0.195]	[0.032, 0.053]	[0.003, 0.005]	[53.629, 70.318]	[0.073, 0.077]	[3.244, 4.872]	[3.747, 9.239]
C11	FE ORF and DE IRF	[0.085, 0.089]	[−0.081, 0.099]	[2.672, 3.084]	[0.227, 0.284]	[0.068, 0.072]	[0.007, 0.008]	[30.872, 36.071]	[0.106, 0.111]	[3.323, 4.022]	[3.895, 4.759]
C12	FE ORF and DE ORF	[0.081, 0.089]	[−0.150, 0.009]	[2.510, 2.961]	[0.221, 0.296]	[0.066, 0.072]	[0.006, 0.008]	[36.222, 37.993]	[0.100, 0.110]	[3.301, 4.154]	[3.855, 4.860]
C13	FE ORF and DE BF	[0.059, 0.061]	[−0.090, 0.074]	[2.637, 2.986]	[0.159, 0.175]	[0.047, 0.050]	[0.004, 0.0043]	[54.090, 67.421]	[0.073, 0.077]	[3.331, 3.920]	[3.874, 4.588]
C14	FE BF and DE IRF	[0.103, 0.107]	[−0.099, 0.079]	[2.474, 2.803]	[0.256, 0.321]	[0.085, 0.089]	[0.011, 0.012]	[24.912, 29.619]	[0.125, 0.131]	[3.062, 3.770]	[3.574, 4.393]
C15	FE BF and DE ORF	[0.098, 0.106]	[−0.155, −0.012]	[2.342, 2.731]	[0.255, 0.231]	[0.082, 0.088]	[0.010, 0.011]	[24.737, 32.152]	[0.118, 0.128]	[3.092, 3.805]	[3.576, 4.517]
C16	FE BF and DE BF	[0.076, 0.080]	[−0.111, 0.054]	[2.408, 2.767]	[0.175, 0.220]	[0.063, 0.067]	[0.007, 0.008]	[47.319, 61.536]	[0.091, 0.096]	[3.083, 3.805]	[3.553, 4.468]

[*a*, *b*] is the close interval between *a* and *b*.

**Table 5:** The GRG and sequence of features extracted.

Number	GRG *ξ* = 0.3	GRG *ξ* = 0.5	GRG *ξ* = 0.6	GRG *ξ* = 1	Features
1	0.912	0.945	0.954	0.972	Root mean sq. value
2	0.912	0.945	0.953	0.971	Standard deviation
3	0.909	0.943	0.952	0.970	Abs. mean value
4	0.868	0.916	0.929	0.956	Skewness
5	0.864	0.910	0.923	0.951	Max peak value
6	0.761	0.833	0.855	0.907	Shape factor
7	0.746	0.830	0.854	0.904	Kurtosis
8	0.480	0.604	0.646	0.752	Crest factor
9	0.412	0.536	0.580	0.695	Impulse factor
10	0.367	0.488	0.532	0.652	Clearance factor

**Table 6:** Comparison of performance between the AGAs-BPNNs and GRA-AGAs-BPNNs.

Number of dominant features selected	BPNNs topology	Training	Validation	Testing	CPU time
**10 features**	**30-30-30-30-16**	**99.3%**	**91.7%**	**92.4%**	**2905.0**
1 feature	3-3-3-3-16	41.5%	38.8%	36.2%	992.2
3 features	9-9-9-9-16	83.6%	80.4%	80.4%	1116.8
**5 features**	**15-15-15-15-16**	**99.4%**	**97.1%**	**96.7%**	**1279.3**
**7 features**	**21-21-21-21-16**	**100.0%**	**94.6%**	**92.5%**	**1468.2**
1 feature (the 10th feature)	3-3-3-3-16	28.7%	26.7%	26.3%	982.6
2 features (the 1st and the 10th features)	6-6-6-6-16	59.9%	52.9%	55.4%	1074.8

**Table 7:** Percentage of increased accuracy and reducing time compared to AGAs-BPNNs without GA.

Number of dominant features	Training (%)	Validation (%)	Testing (%)	Time reduced (%)
5 features	0.1%	5.4%	4.3%	56%
7 features	0.7%	2.9%	0.1%	49.5%
